# Pharmacokinetic Study of Perioperative Intravenous Ifosfamide

**DOI:** 10.1155/2011/185092

**Published:** 2011-09-21

**Authors:** Kurt Van der Speeten, O. Anthony Stuart, Haile Mahteme, Paul H. Sugarbaker

**Affiliations:** ^1^Department of Surgical Oncology, Hospital East-Limburg, 3600 Genk, Belgium; ^2^Washington Cancer Institute, Washington Hospital Center, Washington, DC 20010, USA; ^3^Division of Surgical Sciences, Department of Surgery, Uppsala University Hospital, 75185 Uppsala, Sweden

## Abstract

The use of cancer chemotherapy and hyperthermia as part of a surgical procedure in the management of patients with peritoneal carcinomatosis has gained prominence in recent years with selected patients showing benefit. Patients with peritoneal surface malignancy following cancer resection were treated with intraperitoneal hyperthermic (41.5–42.5°C) cisplatin and doxorubicin combined with the infusion of systemic ifosfamide chemotherapy. The concentrations of ifosfamide and 4-hydroxyifosfamide were determined in plasma, peritoneal fluid, urine, and when possible, within small tumor nodules less than 1 cm. Plasma concentrations of ifosfamide exceeded peritoneal fluid levels of ifosfamide during the 90 minutes of chemotherapy infusion. Both ifosfamide and 4-hydroxyifosfamide could be recovered from peritoneal tumor nodules throughout the 90 minutes of ifosfamide continuous infusion and exceeded plasma concentrations. 4-Hydroxyifosfamide within peritoneal surface cancer nodules suggested a favorable pharmacologic endpoint in the study of ifosfamide administered in the operating room.

## 1. Introduction

The use of intraoperative systemic chemotherapy to target peritoneal carcinomatosis in patients treated with cytoreductive surgery and hyperthermic intraperitoneal chemotherapy was first explored by Elias and coworkers [1]. With this regimen combining intravenous 5-fluorouracil with intraperitoneal oxaliplatin, they reported a median survival of 62.7 months in a selected group of patients with carcinomatosis from colon cancer [2]. This result was compared to matched control patients treated with modern systemic chemotherapy resulting in a median survival of 23.9 months (*P* < 0.05) [2]. The concept of heat targeting of systemic chemotherapy to cancerous tissue has been used successfully in other disease states. Schlemmer and coworkers in a randomized controlled study determined the response of retroperitoneal sarcoma to systemic ifosfamide with and without the addition of deep hyperthermia [3]. Both the sarcoma response and the survival of patients were improved by adding hyperthermia. 

Ifosfamide may be an important drug for clinical trials that evaluate the possible benefits of heat targeting for chemotherapy. It is one of four drugs that show true heat synergy, with 5 to 10 times the duration of tumor control with 41.5°C heat as compared to normal temperatures [4]. In this paper we build on the data provided by Zylberberg and colleagues on the use of intravenous ifosfamide and intraperitoneal cisplatin for the management of peritoneal surface malignancy, especially for ovarian carcinomatosis and peritoneal mesothelioma [5]. Moderate doses of systemic ifosfamide were used simultaneously with intraperitoneal heat for 90 or 150 minutes. The peritoneal surfaces were maintained at 41.5–42.5°C using a large volume of hyperthermic intraperitoneal chemotherapy solution and an external heater circulator [5–7]. The pharmacology of systemic administered intraoperative ifosfamide and its active metabolite, 4-hydroxyifosfamide, is the focus of this paper. Before a clinical trial with survival as an endpoint can be developed, strong pharmacologic support for this method of treatment is required. The pharmacologic endpoint of these treatment protocols is the demonstration of the active form of this drug, 4-hydroxyifosfamide, within the nodules of the peritoneal carcinomatosis. Also, a precise quantitation of adverse events associated with the management is needed before further applications of this approach can be contemplated.

## 2. Materials and Methods

At the Washington Cancer Institute, 16 patients with peritoneal carcinomatosis were included in this study. Patients on whom these pharmacologic studies were performed had a progression of peritoneal carcinomatosis from gynecologic cancer (4), colon (2), gastric (1), appendix (1), or diffuse malignant peritoneal mesothelioma (8). Institutional Review Board approval was obtained to collect and statistically analyze data. Patient accrual was between July 1, 2007 and July 31, 2009. The goal of cytoreductive surgical treatment was to reduce the intra-abdominal and pelvic tumor volume to a cellular level [6–8]. Following this surgical event, the edges of the abdominal incision were elevated to create a reservoir within the abdominal and pelvic space. A large volume (1.5 liters/m^2^) of chemotherapy solution containing cisplatin (50 mg/m^2^) and doxorubicin (15 mg/m^2^) was instilled. In two patients with prior adverse reaction to cisplatin, mitomycin C at 15 mg/m^2^ was used with doxorubicin. The chemotherapy was administered in a 1.5% dextrose peritoneal dialysis solution and maintained by an external heater circulator (Belmont Medical Devices, Billerica, MA, USA) at 41.5–42°C for 90 minutes. The heated chemotherapy solution was uniformly distributed with the surgeon's double-gloved hand. Fifteen minutes prior to ifosfamide continuous infusion sodium-2-mercaptoethane sulfonate (Mesna) at 260 mg/m^2^ was administered intravenously as a bolus in 100 mL of 0.9% sodium chloride. Mesna was used to protect against hemorrhagic cystitis; it was repeated at 4 and 8 hours. At the initiation of the intraperitoneal chemotherapy treatments, the ifosfamide (1,300 mg/m^2^) in one liter of normal saline was started as a continuous infusion at a constant rate over 90 minutes. Prior to the initiation of all chemotherapy treatments, small samples (each approximately one mL) of the intraperitoneal chemotherapy solution and the ifosfamide solution were taken as reference samples. Also samples of blood, urine, and tumor nodules were taken prior to initiation of chemotherapy treatments. These were used for preparation of quality control samples. After initiation of chemotherapy, samples of blood, peritoneal fluid, and urine were taken at 15-minute intervals for the first 60 minutes and then at 30-minute intervals. In some patients, small tumor nodules (0.5–2 cm) were present on the small bowel or small bowel mesenteries which were marked during cytoreduction. These nodules were harvested at 15-minute intervals during the hyperthermic intraperitoneal chemotherapy using curved Mayo scissors. At the end of the procedure, all tumor nodules had been accounted for. These tumor nodules were histopathologically confirmed as being malignant. In 4 patients, the sample collection continued for an additional hour after the ifosfamide infusion was complete. The volume of urine was monitored at 15-minute intervals. If core temperatures reached 39°C, the heating of the chemotherapy solution was reduced. 

### 2.1. Processing and Storage of Samples Obtained in the Operating Room

All samples collected in the operating room were immediately placed on ice until processing. Processing was as outlined by Kerbusch et al. [9]. Blood and peritoneal fluid samples were centrifuged at 3,000 rpm for 10 minutes. The resulting plasma and supernatant from peritoneal fluid along with urine samples were transferred to screw-capped polypropylene tubes for storage at −20°C. Tissue samples were dried of all surface moisture using absorbent gauze pads and placed in screw-capped polypropylene vials for storage at −20°C. These samples untreated by semicarbazide were subsequently used for ifosfamide analysis.

### 2.2. Semicarbazide Stabilization of 4-Hydroxyifosfamide within Samples Obtained in the Operating Room

Prior to storage a 500-microliter sample of each biological fluid (plasma, peritoneal fluid, and urine) was transferred to a small screw-capped vial containing 50 microliters of a 2 M solution of semicarbazide (pH 7.4). Vials were tightly capped and thoroughly mixed on a Vortex mixer for approximately one minute. For quality control, a 450-microliter sample of plasma taken prior to administration of chemotherapy was treated with 50 microliters of a known concentration of 4-hydroxyifosfamide·semicarbazide (4-OHIF·SCZ) prepared from 4-hydroperoxyifosfamide and semicarbazide [10]. A small nodule (approximately 50 mg) from each tissue sample was accurately weighed and homogenized in 450 microliters of 0.5 M NaH_2_PO_4_ (pH 7.4) in a screw-capped vial. Each homogenate was then treated with 50 microliters of semicarbazide and mixed as with the biological fluid samples. A tissue control sample was prepared by homogenizing a small (50 mg) tumor nodule harvested prior to administration of chemotherapy in 450 microliters of 0.5 M NaH_2_PO_4_ to which 50 microliters of a known concentration of 4-OHIF·SCZ was added. All samples were then stored at −20°C until HPLC analysis. All HPLC analyses were performed within 48 hours of time of storage.

### 2.3. Preparation of Samples for HPLC Analysis

#### 2.3.1. Ifosfamide Assay

Ifosfamide levels were determined using a modified version of the procedure described by Margison et al. [11]. All samples were thawed at room temperature prior to HPLC analysis. Peritoneal fluid and urine samples were diluted appropriately with a mobile phase. A 3x to 5x dilution was necessary for peritoneal fluid samples, while urine dilutions ranged from 5x to 15x. After thorough mixing the resulting solutions were filtered through 0.45 micron syringe filters prior to HPLC injection. 

For plasma samples, a 500-microliter sample was mixed with 500 microliters of 0.1 N sodium hydroxide solution in a screw-capped polypropylene centrifuge tube. A 5-milliliter aliquot of a solution of 25% isopropanol in chloroform was then added, and the mixture was thoroughly mixed on a vortex mixer for approximately 2 minutes. After centrifugation at 3000 rpm for 10 minutes, the upper layer was discarded and the lower layer was carefully removed and transferred to a clean polypropylene centrifuge tube and evaporated to dryness under a stream of N_2_ at 39°C. The resulting residue was dissolved in an appropriate volume of mobile phase (2x to 3x) and filtered through a 0.45-micron syringe prior to HPLC injection. For quality control, a 500-microliter sample of “control” plasma with a known concentration of ifosfamide was included in each assay. 

Tumor nodules were again dried of surface moisture before processing for ifosfamide assay. A small tumor nodule (approximately 100 mg) was accurately weighed then minced in 150 microliters of 0.1 N NaOH before being homogenized in 2.5 mL of 25% isopropyl alcohol in chloroform. The homogenate was transferred to a 15 mL polypropylene centrifuge tube and centrifuged at 3,000 rpm for 10 minutes. The lower organic phase was transferred to a clean polypropylene tube and evaporated with an N_2_ stream as with the plasma, peritoneal fluid, and urine. The residue was dissolved in 1 mL mobile phase and filtered through a 0.45-micron syringe filter. For quality control, a 100 mg tumor nodule taken prior to the administration of ifosfamide was minced in 150 microliters of 0.1 N NaOH which contained a known amount of ifosfamide and processed as above. A 50-microliter aliquot of each sample was injected for HPLC analysis.

#### 2.3.2. 4-Hydroxyifosfamide Assay

4-Hydroxyifosfamide levels were determined in the derivatized samples using a slightly modified version of the procedure described by Kerbusch and colleagues [10]. Briefly, all samples were thawed at room temperature. Peritoneal fluid samples and urine samples were diluted appropriately with the mobile phase and filtered through 0.45-micron syringe filters prior to HPLC injection.

The plasma derivatives and the 4OHIF·SCZ plasma control sample were extracted with a 10x volume of acetonitrile in screw-capped polypropylene centrifuge tubes. After centrifugation the clear acetonitrile extracts were transferred to clean polypropylene tubes and evaporated to dryness under a constant stream of N_2_ at 39°C. The residues were each dissolved in 1 mL of the mobile phase and filtered through a 0.45-micron syringe filter before HPLC injection.

The homogenates from the tumor nodule derivatives along with the 4-OHIF·SCZ control sample were thoroughly mixed again before processing. Each homogenate was extracted with 5 milliliters of ethyl acetate in a screw-capped polypropylene centrifuge tube. After centrifugation the organic supernatant was transferred to a clean polypropylene tube and evaporated to dryness under a steady stream of N_2_ at 39°C. The residue was dissolved in 1 mL of the mobile phase and filtered through a 0.45-micron syringe filter before HPLC injection. A 50-microliter aliquot of each sample was injected for HPLC analysis.

### 2.4. Ifosfamide as an Internal Standard

For each patient a reference sample of ifosfamide was obtained in the operating room directly from the bag of chemotherapy solution. This was used to prepare a standard curve by which all other samples taken from the patient were evaluated. It was also used for the preparation of quality control samples.

### 2.5. HPLC System for Ifosfamide and 4-Hydroxyifosfamide

Briefly, the HPLC system consisted of a Shimadzu LZ7A instrument equipped with the SPD-6AV (UV-VIS) Detector set at 190 nm and AC-R6A Chromatopac Data Processor (Shimadzu Instruments, Columbia, MD, USA). Chromatographic separation was accomplished on a C8 Reversed Phase Column (Varian Incorporated, 25200 Commerce Center Drive, Lake Forest, CA, USA). The mobile phase for ifosfamide consisted of 30% acetonitrile in a 0.01 M phosphate buffer (pH 6.5) at a flow rate of 1.5 mL/min.

Concentrations of 4-hydroxyifosfamide were determined using a modified version of the HPLC system as described by Kerbusch and colleagues [9, 10]. The HPLC system was as described for ifosfamide. The mobile phase consisted of 24% acetonitrile in 0.025 M potassium dihydrogenphosphate with 0.1% triethylamine at a flow rate of 0.9 mL/min. The SPD-6AV (UV-VIS) Detector was set at 230 nm.

#### 2.5.1. Data Analysis and Presentation

The concentration of ifosfamide and 4-hydroxyifosfamide in tumor nodules (C_tumor  nodule_) was determined by dividing the concentration of the drug in the tumor nodule homogenate (micrograms of drug per milliliter of homogenate) by the concentration of tissue in the tumor nodule homogenate (grams of tissue per milliliter of homogenate):

(1) $$\begin{array}{cc} {\text{C}}_{\text{tumor nodule}} & ={\text{micrograms}}_{\left(\text{drug}\right)}\times {\text{milliliters}}_{{\left(\text{homogenate}\right)}^{-1}},\\ & \;\;{\text{grams}}_{\left(\text{tissue}\right)}\times {\text{milliliters}}_{{\left(\text{homogenate}\right)}^{-1}}\\ & ={\text{micrograms}}_{\left(\text{drug}\right)},\\ & {\;\;\text{grams}}_{\left(\text{tissue}\right)}. \end{array}$$

This expression of the drug concentration in tumor nodules represents the closest equivalence to the drug concentration in the biologic fluid samples (peritoneal fluid, plasma, and urine). It is based on the assumption that one gram of tissue has a volume of approximately one cubic centimeter. The micrograms/grams was presented in the figures as micrograms/milliliter.

All data was compiled and analyzed using Microsoft Excel (Microsoft Corporation, Redmond, WA, USA). Data were displayed as mean ± 1 standard deviation. The area under the curve data was determined using GraphPad Prism Software (GraphPad Software Inc., La Jolla, CA, USA).

#### 2.5.2. Adverse Events

The database was prospectively completed on individual patients by physician's assistants (RA and GE) as the hospital discharge summary was being dictated. These records were then reviewed by the senior attending or other attending surgeon. Data were entered into the computer by a nurse. The database was constructed specifically to evaluate patients treated for peritoneal surface malignancy [12]. It consisted of 47 adverse events arranged in 8 categories by organ system. The categories and a list of adverse events that were scored for each patient are listed in Table 1. For each adverse event a grade was assigned. For a grade I adverse event, the diagnosis was established, but no treatment was required. For grade II, the adverse event required medical treatment for a resolution. For grade III, the adverse event was potentially serious but was resolved conservatively, often with an invasive intervention for resolution. This was often a computed tomographic- or ultrasound-guided therapeutic intervention. For grade IV, the adverse event required definitive urgent intervention, often a return to the operating room or to the surgical intensive care unit. A grade V adverse event led to the patient's death.

## 3. Results

### 3.1. Ifosfamide Concentrations in Plasma, Peritoneal Fluid, and Urine

**Figure 1:**
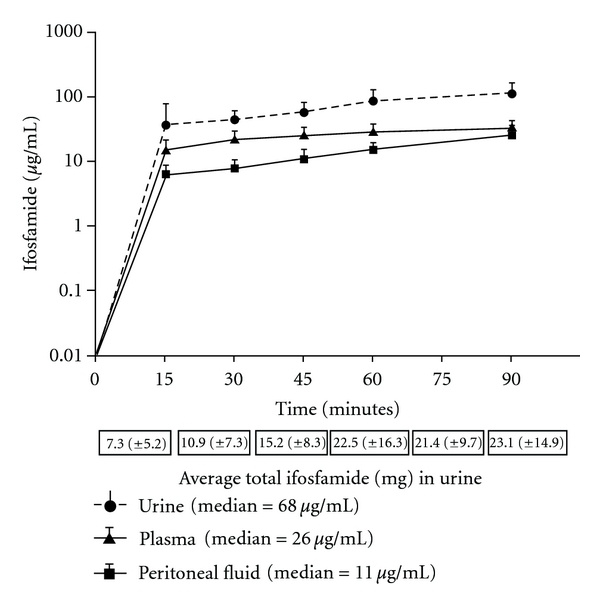
Plasma, peritoneal fluid, and urine concentrations of ifosfamide during a 90-minute continuous infusion. The total quantity of ifosfamide in mg excreted in the urine is presented at the bottom. The median concentration of plasma, peritoneal fluid and urine ifosfamide is shown separately.

Figure 1 shows the ifosfamide concentrations in blood, peritoneal fluid, and urine for 6 patients treated with a 90-minute ifosfamide infusion and 90 minutes of heated intraoperative intraperitoneal chemotherapy (HIPEC). The peritoneal fluid levels rapidly increased; however, in all patients peritoneal fluid remained lower than the plasma ifosfamide levels throughout the 90-minute infusion. A large quantity of the ifosfamide was excreted unchanged in the urine. The median concentration of ifosfamide in plasma, peritoneal fluid, and urine is shown separately; these data are the median values of all concentrations over 90 minutes.

### 3.2. 4-Hydroxyifosfamide Concentrations in Plasma, Peritoneal Fluid, and Urine

**Figure 2:**
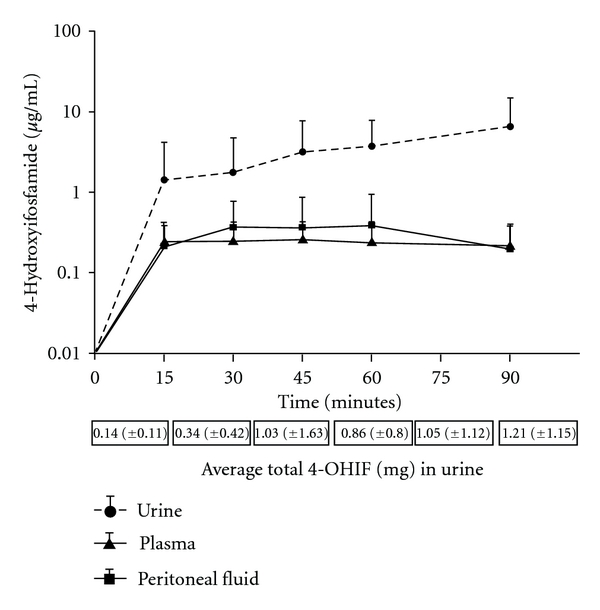
Plasma, peritoneal fluid, and urine concentrations of 4-hydroxyifosfamide over a 90-minute infusion. The total quantity of 4-hydroxyifosfamide in mg excreted in the urine is presented at the bottom.

Figure 2 shows the 4-hydroxyifosfamide concentrations in plasma, peritoneal fluid, and urine for 6 patients treated with a 90-minute ifosfamide infusion and 90 minutes of HIPEC. When a mean concentration of peritoneal fluid and plasma was plotted, the concentrations in these two body compartments continued at similar levels over the 90 minutes. Urine excretion continued to be large for the entire 90 minutes showing that large amounts of the active metabolite of this chemotherapy agent was present unchanged in the urine.

### 3.3. Ifosfamide and 4-Hydroxyifosfamide Concentrations over 150 Minutes

**Figure 3:**
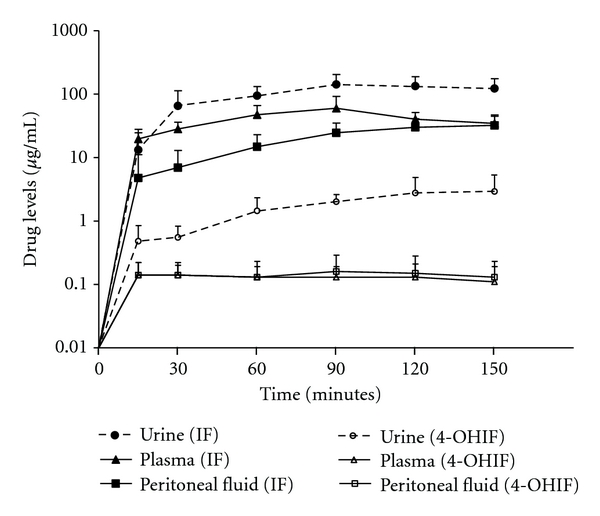
Plasma, peritoneal fluid, and urine concentrations of ifosfamide and 4-hydroxyifosfamide over a 90-minute infusion and 60-minute period of observation.

Figure 3 shows the plasma, peritoneal fluid, and urine concentrations in 10 patients. The ifosfamide continuous infusion was used over the 90 minutes in these patients; however, the volume of intraperitoneal chemotherapy solution and the heating of this chemotherapy solution were maintained for an additional hour for a total of 150 minutes. Elevated concentrations of plasma ifosfamide as compared to peritoneal fluid ifosfamide continued throughout the 90-minute infusion. However, during the 90-minute to 150-minute period, the concentration in the peritoneal fluid and plasma equilibrated.

For 4-hydroxyifosfamide the plasma and peritoneal fluid levels were similar over the 150 minutes. The levels of drug in these two compartments decreased minimally after the infusion ceased at 90 minutes. A prolonged presence of 4-hydroxyifosfamide in the plasma and in the peritoneal fluid is evident.

### 3.4. Comparisons of Ifosfamide and 4-Hydroxyifosfamide Concentrations in Tumor Tissue and in Plasma

**Figure 4:**
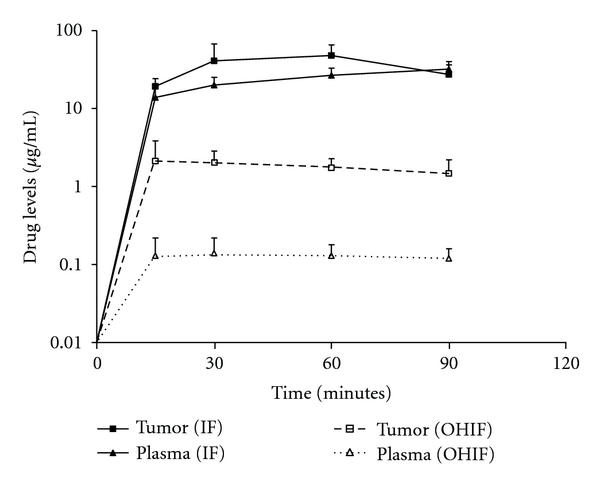
Comparison of ifosfamide and 4-hydroxyifosfamide concentrations in tumor nodules and in plasma.

Figure 4 shows the mean ifosfamide concentration in tumor nodules harvested in 5 patients over 90 minutes. Also shown are the mean concentrations in plasma. The concentrations in tumor nodules were consistently elevated as compared to the concentrations determined within the plasma for 60 minutes. Similar data were obtained in 4 of these 5 patients for 4-hydroxyifosfamide. Levels of 4-hydroxyifosfamide in tumor tissues were consistently greater than observed within the plasma.

### 3.5. Four- and Eight-Hour Measurements of Ifosfamide and 4-Hydroxyifosfamide

In 3 patients, blood and urine samples were obtained at 4 and 8 hours after initiation of the ifosfamide infusion. The mean ifosfamide concentration in the plasma at 4 hours and 8 hours, respectively, was 27.4 ± 2.4 *μ*g/mL and 22.02 ± 0.8 *μ*g/mL. The mean concentration in the urine at 4 hours and 8 hours, respectively, was 138.0 ± 85.7 *μ*g/mL and 58.45 ± 35.1 *μ*g/mL. Considerable quantities of active drug remain in the plasma and urine of these patients 8 hours following infusion of the chemotherapy. The four-hour concentrations of 4-hydroxyifosfamide in plasma and urine were also determined. The mean 4-hydroxyifosfamide concentration in plasma at 4 hours was 0.06 ± 0.03 *μ*g/mL. The mean 4-hydroxyifosfamide in urine at 4 hours was 3.4 ± 2.1 *μ*g/mL.

### 3.6. Adverse Events

The clinical features on these 16 patients treated with cytoreductive surgery and perioperative hyperthermic chemotherapy are listed in Table 2. There were no abnormalities of BUN or creatinine prior to or after the bidirectional chemotherapy treatments neither was hematuria observed. Table 2 itemizes the adverse events that were prospectively recorded. There were no mortalities, two grade IV adverse events (13%), and no grade III events.

## 4. Discussion

### 4.1. Heat Targeting of Cancer Chemotherapy

Two decades of research seeking the mechanisms for heat augmentation of chemotherapy killing have been published [4, 13–21]. It is possible that improvements in the care of cancer patients result with the concomitant use of heat and systemic chemotherapy. Unfortunately, logistical problems with the simultaneous administration of heat and chemotherapy occur in most clinical situations. Schlemmer and colleagues were able to overcome these logistical problems and combined intravenous doxorubicin and ifosfamide with deep hyperthermia for truncal sarcoma [3]. In a randomized study they showed improved responses when heat was added to the intravenous infusion of systemic chemotherapy. Several groups showed that the intravesical instillation of mitomycin C was more effective when the chemotherapy solution in the bladder was maintained at 43°C as compared to body temperature [21]. In the present study, systemic ifosfamide was targeted to the peritoneal surface cancer nodules by flooding the peritoneal cavity with a large volume of heated peritoneal dialysis solution. Small tumor nodules were heated to at least 41.5°C. Larger tumor nodules would be more resistant to heat penetration of the entire mass of cancer. Using this technology the cytotoxic effects of ifosfamide are expected to be maximized within heated peritoneal surface tumor nodules.

### 4.2. Rationale Supporting Bidirectional Intraoperative Chemotherapy

In this treatment strategy the bidirectional (intraperitoneal and intravenous) administration of cancer chemotherapy may provide a unique advantage for treatment of carcinomatosis. The chemotherapy solution within the abdomen contains cisplatin and doxorubicin. By simple diffusion these two chemotherapy agents would enter the tissue of small cancer nodules. High levels of doxorubicin and cisplatin have been documented within peritoneal surface cancer nodules [22, 23]. However, not only are these intraperitoneal chemotherapy agents augmented by heat, but also the 4-hydroxyifosfamide is heated as it reaches the tumor nodule from the bloodstream. Chemotherapy from both the intraperitoneal and intravenous compartments converges on the tissues at the interface of heated intraperitoneal fluid and peritoneal surface. These are the tissues that contain peritoneal carcinomatosis or peritoneal mesothelioma nodules.

### 4.3. Ifosfamide as a Prodrug

Ifosfamide is a difficult drug to study pharmacologically. Its anticancer effects only occur after the prodrug, ifosfamide, is metabolized in the liver and red blood cells by the cytochrome system to 4-hydroxyifosfamide. The active metabolite is unstable and only exists for a few minutes within the plasma or within the red blood cell. The larger standard deviations in 4-hydroxyifosfamide levels in Figure 2 as compared to the data of Figure 1 with ifosfamide may result from this instability. The activity of ifosfamide cannot be studied in vitro because the drug is not activated in the absence of metabolism. This fact makes our pharmacologic in vivo studies of ifosfamide even more important. We have been able to document the presence of the active compound within the peritoneal fluid bathing small cancer nodules. We have also been able to show that the active molecule, 4-hydroxyifosfamide, is present within these small tumor nodules. This quantitation of 4-hydroxyifosfamide is possible as a result of the stabilization of the molecule with semicarbazide [9, 10].

### 4.4. Choice of Intravenous Ifosfamide to Augment Hyperthermic Intraperitoneal Chemotherapy

Ifosfamide is a broadly active chemotherapy highly active in soft tissue sarcoma, nonsmall cell lung cancer, and ovarian cancer [24]. Its unique activity for use along with heated chemotherapy solutions comes from a remarkable augmentation of cytotoxicity by moderate heat. Urano et al. in a mouse foot-pad model demonstrate a tumor growth rate to decrease by one-third when ifosfamide was used at 41.5°C as opposed to room temperature [4]. In small tumor nodules that remain after cytoreduction intraperitoneal heat should cause increased cytotoxicity at the peritoneal surface in the absence of increased systemic toxicity.

### 4.5. Ifosfamide as a Continuous Infusion

The ifosfamide in this study is delivered as a continuous infusion, and, therefore, these data are different than our previous publications using systemic 5-fluorouracil [25]. With 5-fluorouracil the drug is administered as a bolus over 7 minutes. The blood and peritoneal fluid equilibrate within 15 minutes. However, after equilibration, drug levels within the peritoneal fluid remain at a higher level than those in the plasma. If a pharmacologic analysis of ifosfamide is performed after the 90 minutes of continuous infusion, the same phenomenon of more rapid metabolism in the peripheral blood as compared to the peritoneal space is observed. The phenomenon does not occur nearly so rapidly because the metabolism of ifosfamide is slower than 5-fluorouracil. These data show that the method of administration of the intravenous drug (bolus as compared to continuous infusion) may have a profound effect on the pharmacokinetics parameters measured in this perioperative use of cancer chemotherapy.

### 4.6. Comparison of Plasma and Peritoneal Fluid Ifosfamide and 4-Hydroxyifosfamide Concentrations

The plasma levels of ifosfamide are always greater than the peritoneal fluid levels. Perhaps this is caused by the continuous drug infusion into the venous blood and its delay in distribution to the peritoneal space. This would account for the persistent higher levels of ifosfamide within the 90-minute infusion. However, during the 90-minute to 150-minute period, the concentration in the peritoneal fluid and plasma equilibrated. 

For 4-hydroxyifosfamide, the variability of levels in the peritoneal fluid versus plasma was evident by the wide standard deviations calculated. However, a prolonged presence of 4-hydroxyifosfamide in the plasma and in the peritoneal fluid was evident.

### 4.7. Morbidity and Mortality

The morbidity and mortality of this treatment plan are presented in Table 2. It compares favorably with the morbidity and mortality previously described for cytoreductive surgery with hyperthermic intraperitoneal chemotherapy [26–28]. Previous studies have documented that the major factor influencing morbidity with cytoreductive surgery and perioperative hyperthermic chemotherapy is the extent of surgery [29, 30]. In these two patients the grade IV morbidity was related to the disease process rather than the chemotherapy toxicities.

### 4.8. Prolonged Hyperthermia to Maximize Chemotherapy Effect

After the first seven patients received a 90-minute infusion of ifosfamide and 90 minutes of intraperitoneal hyperthermia, we observed, by taking blood samples at 2.5 hours after the initiation of infusion, that high levels of ifosfamide and 4-hydroxyifosfamide were maintained. It became evident that prolonging the hyperthermia within the peritoneal space would continue the cytotoxic effects of the cancer chemotherapy without any increase in its systemic toxicity. Therefore, a 2.5-hour hyperthermia treatment to maximize the effects of heat should be considered to maximize cytotoxicity.

### 4.9. Variability of Plasma and Peritoneal Fluid Concentrations of 4-Hydroxyifosfamide

In the monitoring of these patients, the concentrations over 90 minutes of plasma ifosfamide were always slightly increased over the peritoneal fluid ifosfamide concentrations. This observation occurred in all 11 patients. However, with 4-hydroxyifosfamide, considerable variation in the levels of plasma and peritoneal fluid hydroxyifosfamide occurred. Sometimes the plasma 4-hydroxyifosfamide was considerably greater than that found in the peritoneal fluid. In other patients the reverse was seen. Individual patient variations may account for these differences including variable metabolism of ifosfamide to 4-hydroxyifosfamide, variable ascites accumulation during the 90-minute infusion, and variable urine output of 4-hydroxyifosfamide.

### 4.10. Long-Term Activity of Ifosfamide and 4-Hydroxyifosfamide

In this study, in four patients we documented the high levels of ifosfamide and the active molecule 4-hydroxyifosfamide present long term in the blood of these patients. This observation attests to the need for Mesna over a long-time period when using ifosfamide.

### 4.11. Analogies to the Zylberberg Protocol

The treatment strategy that has been employed in our studies is very similar to that published by Zylberberg and colleagues for ovarian cancer [5]. His study showed excellent clinical results when systemic ifosfamide infusion was combined with intraperitoneal cisplatin. We modified his ifosfamide regimen using an infusion over 90 minutes in the operating room. The results that he has achieved with this bidirectional chemotherapy administration (intravenous and intraperitoneal) at normothermic temperatures may be the best long-term results with ovarian malignancy reported to date. Our group is hopeful that modification of his treatment strategy through the use of heat targeting of the cytotoxic effects of 4-hydroxyifosfamide to peritoneal surfaces may further benefit patients with peritoneal carcinomatosis and peritoneal mesothelioma.

### 4.12. Pharmacologic Limitations of the Current Study

Although the ifosfamide levels in blood, peritoneal fluid, and urine could be verified through the use of a blank, this was not true in the determination of 4-hydroxyifosfamide levels. No standard molecule was available to add to normal blood, peritoneal fluid, and urine that would verify our HPLC technology. The fact that 4-hydroxyifosfamide was documented to be present within the cancer nodules is, in and of itself, an important observation.

### 4.13. Clinical Limitations of the Current Study

These data show a pharmacologic rationale for ifosfamide as a reasonable addition to hyperthermic intraperitoneal chemotherapy in an attempt to improve the effects of the perioperative treatments. However, this pharmacologic rationale should be substantiated in clinical trials supporting the survival benefit of this pharmacologically engineered treatment protocols. A phase II study of the individual diseases appropriate for this approach needs to occur. The obvious choices for these studies are stage III ovarian cancer and peritoneal mesothelioma. Also, patients with sarcomatosis or a high risk of sarcomatosis after primary cancer resection could be considered for a treatment protocol similar to that used in this study. Of course, if phase II data is favorable and the morbidity and mortality stay within an acceptable limit, prospective and randomized studies of patients with peritoneal metastases from ovarian cancer and sarcoma should be considered; also, peritoneal mesothelioma is an ideal clinical setting.

## 5. Conclusion

A clear understanding of the pharmacology of perioperative intraperitoneal hyperthermic chemotherapy combined with systemic chemotherapy in the operating room may provide important information for the design of an optimal treatment for peritoneal surface malignancy. These data showed that a systemic drug, ifosfamide, was present in the peritoneal fluid and in the tumor nodules in patients with peritoneal carcinomatosis or peritoneal mesothelioma. These small nodules in direct contact with the hyperthermic intraperitoneal chemotherapy solution may have marked augmentation of the cytotoxicity of the systemic chemotherapy. Ifosfamide may be an ideal drug for systemic administration with local-regional heat because it is greatly augmented in its effects by moderate heat. Application of these treatments in ovarian malignancy and peritoneal mesothelioma may be of benefit.

##  Conflict of Interests

The authors declare that they have no conflict of interests relating to the publication of this paper.

## Figures and Tables

**Table 1:** Postoperative morbidity-mortality database in patients with peritoneal surface malignancy treated by cytoreductive surgery, hyperthermic intraperitoneal cisplatin and doxorubicin, and simultaneous intravenous ifosfamide. Eight categories of events and 47 adverse outcomes were scored.

Category	Adverse event
Gastrointestinal	Anastomotic failure
	Fistula
	Pancreatic fistula
	Pancreatitis
	Bile leak from liver surface
	Hartmann pouch blowout
	Enterostomy tube complication
	Oral pain/ulceration
	Nausea/vomiting
	Diarrhea
	Ascites
	Other
Pulmonary	Respiratory distress
	Pleural effusion
	Pneumonia
	ARDS
	Chest tube removal
	Other
Intravenous catheter status	Line sepsis
	Line thrombosis
	Insertion pneumothorax
	Total parenteral nutrition intolerance
	Other
Cardiovascular	Rhythm
	Hypotension
	Ischemia
	Pulmonary embolism
	Thrombophlebitis
	Venous thrombosis
	Pulmonary edema
	5-Fluorouracil cardiac toxicity
	Other
Genitourinary	Urinary tract infection
	Urine leak
	Other
Hematological	WBC
	Platelets
	Postoperative hemorrhage
	Units of fresh-frozen plasma
	Other
Neurotoxicity	State of communication
	Orientation/intellect
	Stroke
	Other
Infection	Intra-abdominal infection
	Wound infection
	Other

ARDS: acute respiratory distress syndrome; WBC: white blood cell count.

**Table 2:** Clinical features and prospective morbidity-mortality assessment of 16 patients with peritoneal surface malignancy having cytoreductive surgery and perioperative hyperthermic chemotherapy.

Patient	Gender/age	Diagnosis	Grade II morbidity	Grade III morbidity	Grade IV morbidity	Mortality	Current status (months)
1	M/47	Appendix	0	0	0	0	DOD (9)
2	F/56	Papillary serous	0	0	0	0	AWD (26)
3	F/44	Papillary serous	0	0	Hyponatremia, return to SICU	0	NED (31)
4	F/55	Papillary serous	0	0	0	0	NED (29)
5	M/19	Recurrent colon	Postoperative bleeding	0	0	0	AWD (19)
6	M/54	DMPM	Central line infection	0	0	0	AWD (20)
7	F/30	DMPM	Urinary tract infection	0	Excessive ascites, return to SICU	0	AWD (18)
8	F/61	Endometrial	Urinary tract infection	0	0	0	DOD (6)
9	F/60	DMPM	Pleural effusion	0	0	0	AWD (18)
10	F/51	DMPM	0	0	0	0	NED (16)
11	M/54	DMPM	Neuropraxia	0	0	0	NED (14)
12	M/47	Colon	0	0	0	0	AWD (14)
13	M/61	DMPM	0	0	0	0	AWD (14)
14	M/64	Gastric	0	0	0	0	DOD (6)
15	M/52	DMPM	0	0	0	0	NED (13)
16	M/58	DMPM	0	0	0	0	AWD (12)

DMPM: diffuse malignant peritoneal mesothelioma; DOD: died of disease; AWD: alive with disease; NED: no evidence of disease; SICU: surgical intensive care unit.

## References

[1] Elias D, Bonnay M, Puizillou JM (2002). Heated intra-operative intraperitoneal oxaliplatin after complete resection of peritoneal carcinomatosis: pharmacokinetics and tissue distribution. *Annals of Oncology*.

[2] Elias D, Lefevre JH, Chevalier J (2009). Complete cytoreductive surgery plus intraperitoneal chemohyperthermia with oxaliplatin for peritoneal carcinomatosis of colorectal origin. *Journal of Clinical Oncology*.

[3] Schlemmer M, Wendtner CM, Issels RD (2003). Ifosfamide with regional hyperthermia in soft-tissue sarcomas. *Oncology*.

[4] Urano M, Kuroda M, Nishimura Y (1999). For the clinical application of thermochemotherapy given at mild temperatures. *International Journal of Hyperthermia*.

[5] Zylberberg B, Dormont D, Madelenat P, Daraï E (2004). First-line intraperitoneal cisplatin-paclitaxel and intravenous ifosfamide in Stage IIIc ovarian epithelial cancer. *European Journal of Gynaecological Oncology*.

[6] Sarnaik AA, Sussman JJ, Ahmad SA, Lowy AM (2003). Technology of intraperitoneal chemotherapy administration: a survey of techniques with a review of morbidity and mortality. *Surgical Oncology Clinics of North America*.

[7] Dahlke MH, Schlitt HJ, Piso P (2007). Continuous peritoneal perfusion: techniques, methods and applications. *Cancer Treatment and Research*.

[8] Esquivel J (2009). Technology of hyperthermic intraperitoneal chemotherapy in the United States, Europe, China, Japan, and Korea. *Cancer Journal*.

[9] Kerbusch T, De Kraker J, Keizer HJ (2001). Clinical pharmacokinetics and pharmacodynamics of ifosfamide and its metabolites. *Clinical Pharmacokinetics*.

[10] Kerbusch T, Huitema ADR, Kettenes-Van Den Bosch JJ (1998). High-performance liquid chromatographic determination of stabilized 4-hydroxyifosfamide in human plasma and erythrocytes. *Journal of Chromatography B*.

[11] Margison JM, Wilkinson PM, Cerny T, Thatcher N (1986). A simple quantitative HPLC assay for ifosfamide in biological fluids. *Biomedical Chromatography*.

[12] Sugarbaker PH, Alderman R, Edwards G (2006). Prospective morbidity and mortality assessment of cytoreductive surgery plus perioperative intraperitoneal chemotherapy to treat peritoneal dissemination of appendiceal mucinous malignancy. *Annals of Surgical Oncology*.

[13] Sticca RP, Dach BW (2003). Rationale for hyperthermia with intraoperative intraperitoneal chemotherapy agents. *Surgical Oncology Clinics of North America*.

[14] Dahl O, Dalene R, Schem BC, Mella O (1999). Status of clinical hyperthermia. *Acta Oncologica*.

[15] Sugarbaker PH (2007). Laboratory and clinical basis for hyperthermia as a component of intracavitary chemotherapy. *International Journal of Hyperthermia*.

[16] Lepock JR, Borrelli MJ (2005). How do cells respond to their thermal environment?. *International Journal of Hyperthermia*.

[17] Kampinga HH (2006). Cell biological effects of hyperthermia alone or combined with radiation or drugs: a short introduction to newcomers in the field. *International Journal of Hyperthermia*.

[18] Hahn GM, Braun J, Har Kedar I (1975). Thermochemotherapy: synergism between hyperthermia (42-43°) and adriamycin (or bleomycin) in mammalian cell inactivation. *Proceedings of the National Academy of Sciences of the United States of America*.

[19] Kusumoto T, Holden SA, Ara G, Teicher BA (1995). Hyperthermia and platinum complexes: time between treatments and synergy in vitro and in vivo. *International Journal of Hyperthermia*.

[20] Barlogie B, Corry PM, Drewinko B (1980). In vitro thermochemotherapy of human colon cancer cells with cis-dichlorodiammineplatinum(II) and mitomycin C. *Cancer Research*.

[21] Colombo R (2008). Combined treatment with local thermo-chemotherapy for non muscle invasive bladder cancer. the present role in the light of acquired data and preliminary cumulative clinical experiences. *Archivio Italiano di Urologia e Andrologia*.

[22] Jacquet P, Averbach A, Stuart OA, Chang D, Sugarbaker PH (1998). Hyperthermic intraperitoneal doxorubicin: pharmacokinetics, metabolism, and tissue distribution in a rat model. *Cancer Chemotherapy and Pharmacology*.

[23] Los G, Mutsaers PHA, Lenglet WJM, Baldew GS, McVie JG (1990). Platinum distribution in intraperitoneal tumors after intraperitoneal cisplatin treatment. *Cancer Chemotherapy and Pharmacology*.

[24] Dechant KL, Brogden RN, Pilkington T, Faulds D (1991). Ifosfamide/Mesna. A review of its antineoplastic activity, pharmacokinetic properties and therapeutic efficacy in cancer. *Drugs*.

[25] Van Der Speeten K, Stuart OA, Mahteme H, Sugarbaker PH (2010). Pharmacology of perioperative 5-fluorouracil. *Journal of Surgical Oncology*.

[26] Jacquet P, Stephens AD, Averbach AM (1996). Analysis of morbidity and mortality in 60 patients with peritoneal carcinomatosis treated by cytoreductive surgery and heated intraoperative intraperitoneal chemotherapy. *Cancer*.

[27] Mohamed F, Moran BJ (2009). Morbidity and mortality with cytoreductive surgery and intraperitoneal chemotherapy: the importance of a learning curve. *Cancer Journal*.

[28] Smeenk RM, Verwaal VJ, Zoetmulder FAN (2007). Learning curve of combined modality treatment in peritoneal surface disease. *British Journal of Surgery*.

[29] Elias D, Gilly F, Boutitie F (2010). Peritoneal colorectal carcinomatosis treated with surgery and perioperative intraperitoneal chemotherapy: retrospective analysis of 523 patients from a multicentric french study. *Journal of Clinical Oncology*.

[30] Sugarbaker PH, Jablonski KA (1995). Prognostic features of 51 colorectal and 130 appendiceal cancer patients with peritoneal carcinomatosis treated by cytoreductive surgery and intraperitoneal chemotherapy. *Annals of Surgery*.

